# Supplementary material for: Extended intergenic DNA contributes to neuron-specific expression of neighboring genes in the mammalian nervous system
Source: Nat Commun. 2022 May 18;13:2733. doi: 10.1038/s41467-022-30192-z (PMC9117226; doi:10.1038/s41467-022-30192-z)
Supplement: Supplementary file 1 — Supplementary Info [file 41467_2022_30192_MOESM1_ESM.pdf]

Extended intergenic DNA contributes to neuron-specific expression of neighboring genes in the mammalian nervous system

Cell/Tissue	# of intergenic enhancers involved in enhancer-TSS interactions of EID genes	# of genes involved in these interactions	# of enhancers interacting with both neighboring genes	# of enhancers interacting with only one neighboring gene
Cortical neurons	1,254	818	57	905
Cortex	13,142	2,539	259	7,929
Hippocampus	4,043	1,298	46	2,959

Supplementary Table 1 Most enhancers in long intergenic DNA interact with one neighboring gene in neural tissues.

Hi-C datasets of mouse cortical neurons, the human cortex, and the human hippocampus were used to examine long-range chromatin interactions (Supplementary Data 1). Only interactions between intergenic enhancers and transcription start sites (TSSs) of genes with extremely long intergenic DNA (>500 kb, EID genes) were considered to calculate the number (#) of intergenic enhancers in EID genes (Supplementary Data 15).

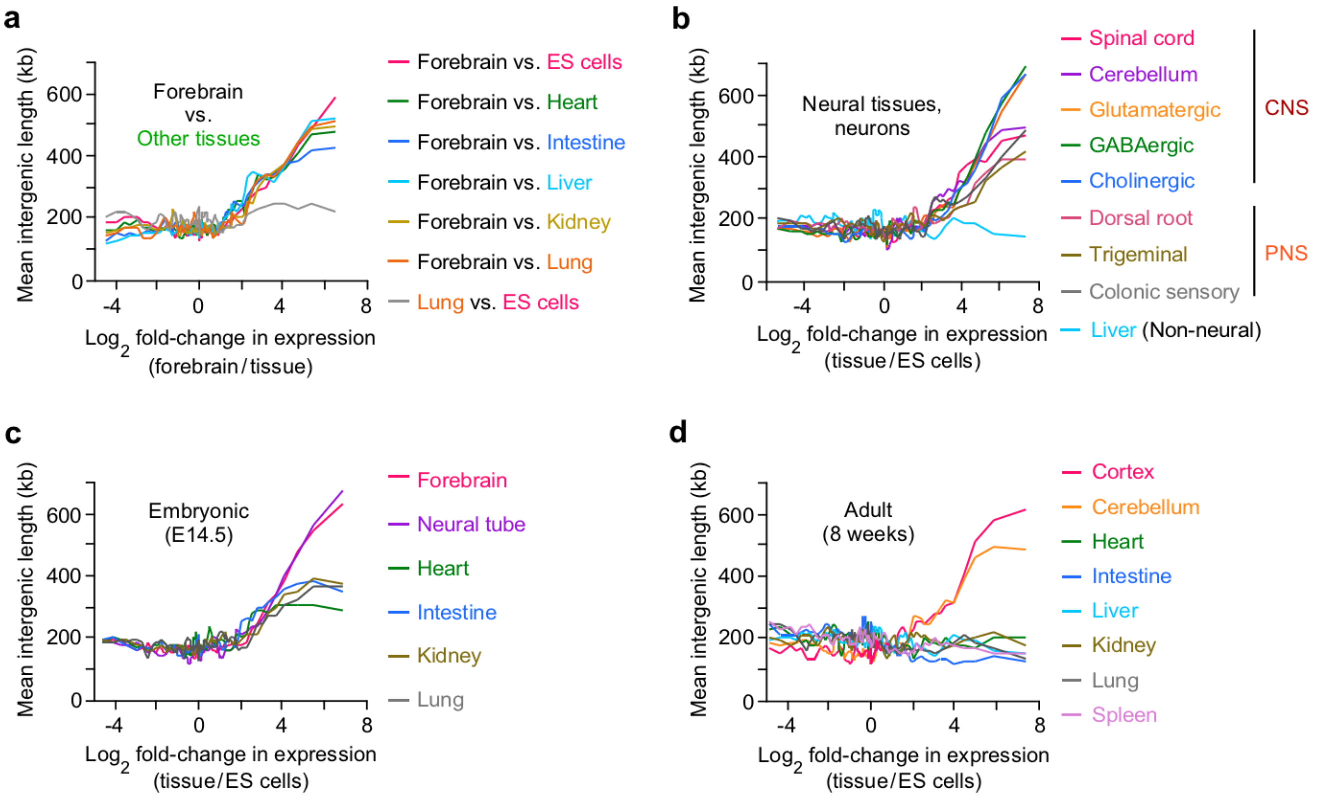

Supplementary Fig 1 Highly induced genes in mouse neural tissues have extended intergenic DNA compared to non-neural tissues and cells in the mouse embryonic and adult stages.

a Genome-wide relationship between intergenic DNA length and gene induction level for the postnatal (P0) forebrain in mice. The lines represent the mean intergenic length for genes binned according to the gene induction level for the forebrain (P0) compared to (vs.) other postnatal (P0) tissues or ES cells ($\log_2$ fold-change in FPKM, 200 genes per bin, 18,383 genes) (Supplementary Methods).

b Genome-wide intergenic DNA length for genes binned according to the gene induction level for adult mouse tissues or neurons, compared to ES cells ($\log_2$ fold-change in FPKM tissue/ES cells). Glutamatergic (Vglut1-positive) and GABAergic (Vgat-positive) neurons indicate excitatory and inhibitory neurons in the central nervous system (CNS), respectively. Trigeminal ganglia, dorsal root neurons (Advillin-positive), and colonic neurons are sensory neurons in the peripheral nervous system (PNS). The lines represent the mean intergenic length for genes within each bin for all the mRNA genes (200 genes per bin, 18,383 genes).

c, d Genome-wide intergenic DNA length for genes binned according to the gene induction level for each mouse E14.5 embryonic (**c**) and 8-week adult tissue (**d**), compared to ES cells ($\log_2$ fold-change in FPKM tissue/ES cells). The lines represent the mean intergenic length for genes within each bin for all the mRNA genes (200 genes per bin, 18,383 genes). The FPKM values of all the mRNA genes in mouse tissues used in (**a**, **b**, **c**) are listed in Supplementary Data 2.

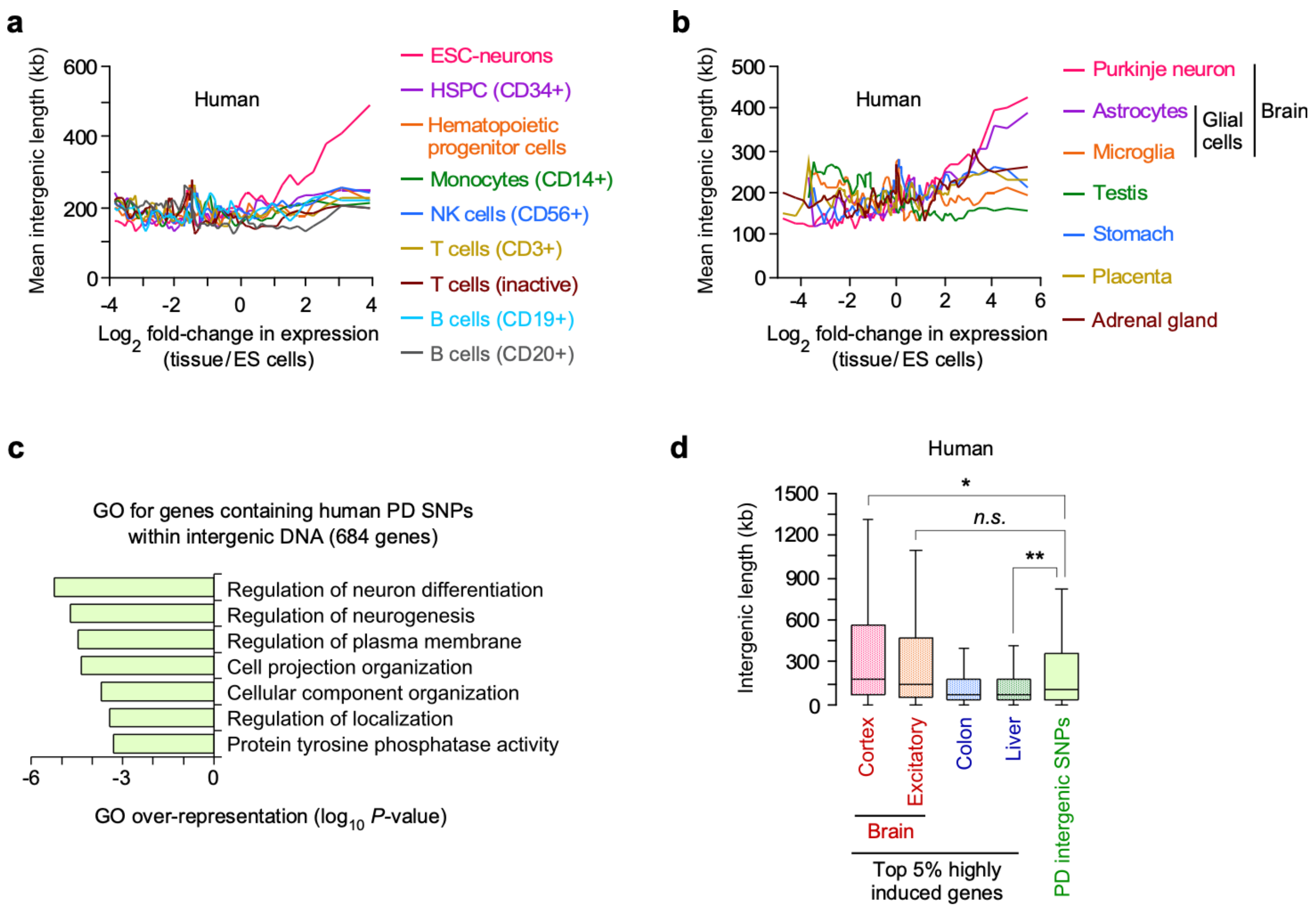

Supplementary Fig 2 Highly induced genes in human neural tissues have extended intergenic DNA.

a Genome-wide intergenic DNA length for genes binned according to the gene induction level for human neurons and hematopoietic cell types compared to human ES cells ($\log_2$ FPKM tissue/ES cells). The lines represent the mean intergenic length for genes within each bin for all the mRNA genes (200 genes per bin, 19,268 genes). Information of each cell type is listed in Supplementary Data 1.

b Genome-wide intergenic DNA length for genes binned according to the gene induction level for each human tissue or ES cell-derived neuron compared to human ES cells ($\log_2$ FPKM tissue/ES cells). The lines represent the mean intergenic length for genes within each bin for all the mRNA genes (200 genes per bin, 19,268 genes, Supplementary Data 4).

c The top seven non-redundant gene ontology (GO) terms for the genes containing high-risk SNPs of Parkinson's Disease (PD) within their intergenic DNA regions (684 genes). The most enriched seven non-redundant GO terms are shown. A one-sided Fisher's exact test was used to calculate *P*-values in gene set enrichment analysis. *P*-values were not adjusted for multiple comparisons.

d Box plot of intergenic DNA lengths of the top 5% of all the mRNA genes, highly induced in the human cortex and excitatory neurons shown in **Fig. 1b** (963 genes, Supplementary Data 4), and the genes containing high-risk SNPs of PD (684 genes). The box plots show the median (line in a box), first-to-third quartiles (boxes) and 1.5 × the interquartile range (whiskers) $^{**}P = 5.32 \times 10^{-11}$; $^{*}P = 0.029$; *n.s.*, non-significant $P = 0.78$; two-sided, two-sample *t*-test.

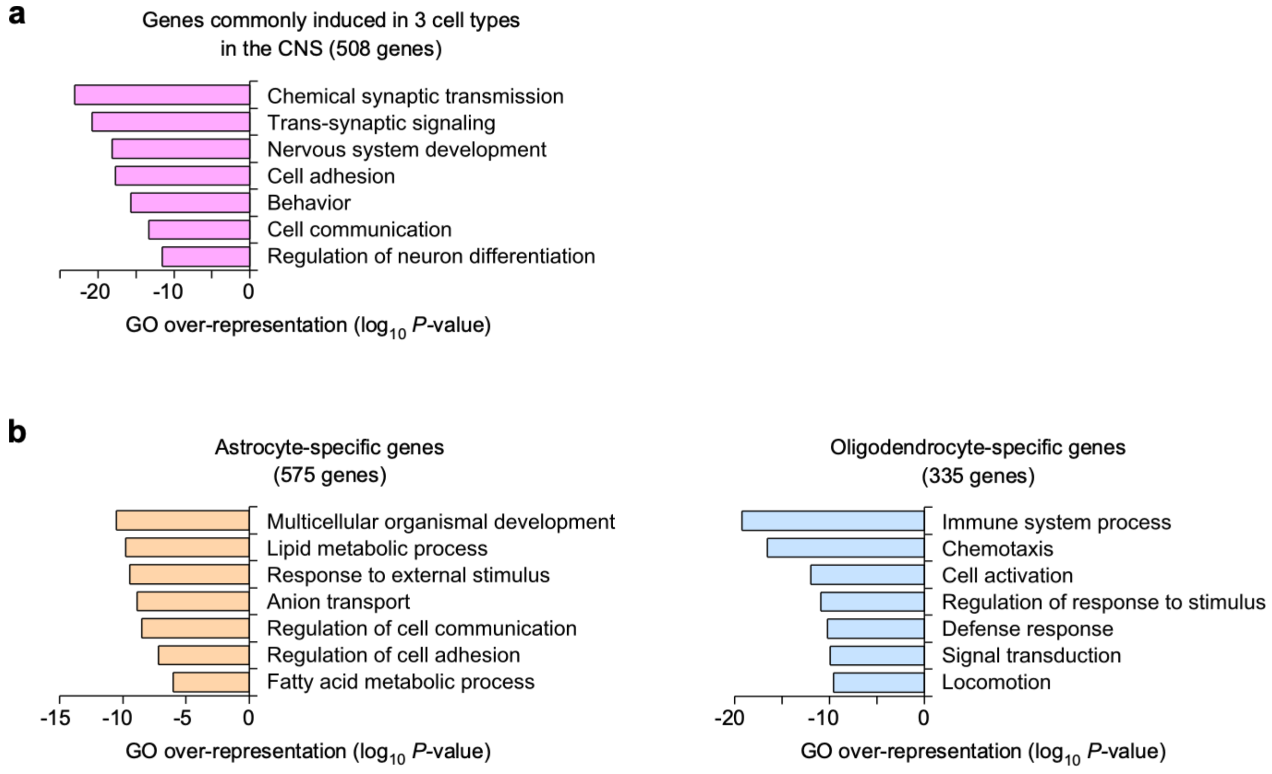

Supplementary Fig 3 Gene highly induced in all 3 CNS cell types are enriched with neural gene ontologies.

a, b Gene ontology analysis of the genes (508) commonly induced in 3 CNS cell types (glutamatergic neurons, astrocytes, and oligodendrocytes), astrocyte-specific genes (575), and oligodendrocyte-specific genes (335) defined in **Fig. 3c**. The most enriched seven non-redundant GO terms are shown. A one-sided Fisher's exact test was used to calculate *P*-values in gene set enrichment analysis. *P*-values were not adjusted for multiple comparisons. All GO target genes and background genes are listed in Supplementary Data 6.

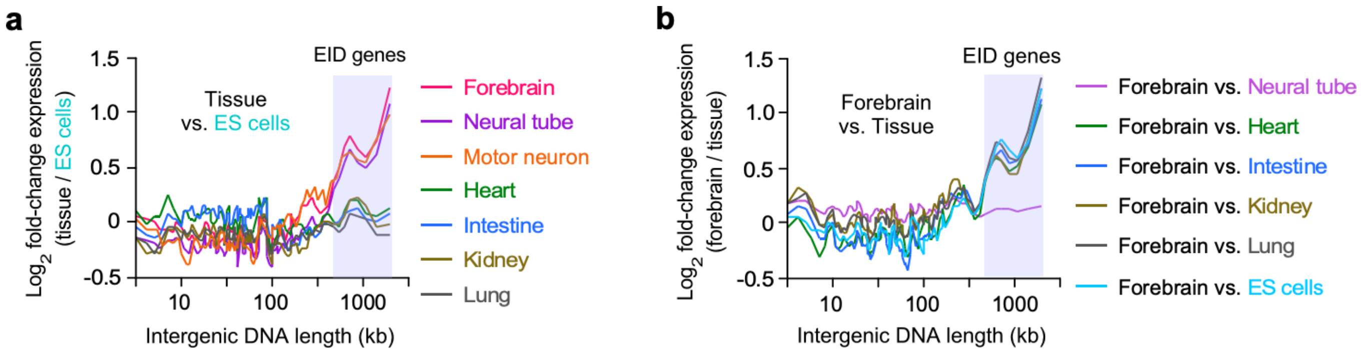

Supplementary Fig 4 Genes with extended intergenic DNA are highly induced in neural tissues compared to non-neural tissues and ES cells.

a Genome-wide gene induction level for each mouse tissue (P0) compared to (vs.) ES cells (log_2 fold-change in FPKM tissue/ES cells), which were binned according to intergenic length (200 genes per bin, 18,383 genes). Genes with extremely long intergenic DNA (>500 kb, EID genes; shaded) are highly induced in neural tissues (Supplementary Data 2).

b Genome-wide gene induction level for the postnatal (P0) mouse forebrain compared to (vs.) non-neural tissues (P0) or ES cells (log_2 fold-change in FPKM forebrain/other tissue), which were binned according to the intergenic length (200 genes per bin, 18,383 genes). The lines represent the mean gene induction level within each bin for all the mRNA genes. Genes highly induced in the forebrain, compared to not only embryonic stem (ES) cells but also non-neural tissues, had extended intergenic DNA.

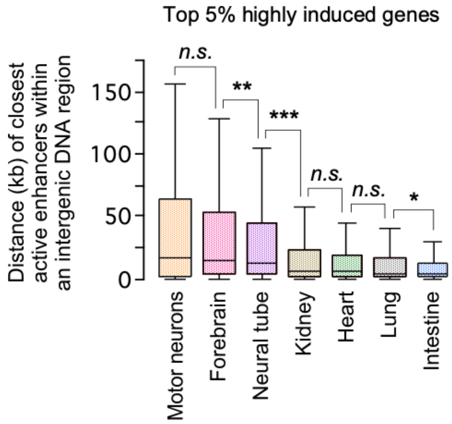

Supplementary Fig 5 Enhancers associated with neural genes are more likely to be dispersed throughout extended intergenic regions.

Box plots of the distance of the closest enhancers within an intergenic DNA region (kb) of the top 5% of all the mRNA genes (919 out of 18,383), highly induced in each tissue in the postnatal P0 mice. Active enhancers were defined as ChIP-seq H3K27ac-enriched regions described in **Fig. 4**. The box plots show the median (line in a box), first-to-third quartiles (boxes), and 1.5 × the interquartile range (whiskers). ***$P = 3.16 \times 10^{-12}$; **$P = 1.36 \times 10^{-2}$; *$P = 7.24 \times 10^{-2}$; *n.s.*, non-significant $P > 0.15$; two-sided, two-sample *t*-test (Supplementary Data 8).

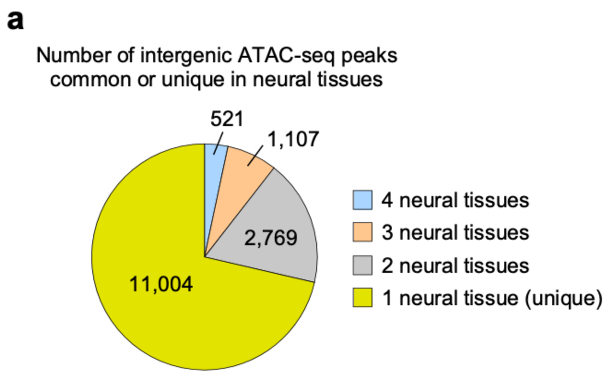

Supplementary Fig 6 The majority of long intergenic regions of generic neural genes have distinct tissue-specific ATAC-seq peaks in multiple neural tissues.

a Pie chart of the number of intergenic ATAC-seq peaks of 625 generic neural genes with >100 kb intergenic regions, grouped by commonly accessible DNA sites in 2-4 neural tissues and tissue-specific accessible DNA sites. **Fig. 5b** shows 521 commonly accessible DNA sites and 11,004 tissue-specific accessible DNA sites. The majority of the intergenic ATAC-seq peaks (11,004 out of 15,401) are distinct in each neural tissue (Supplementary Data 5).

b A pie chart of the number of intergenic regions of 625 generic neural genes having distinct ATAC-seq peaks in multiple tissues. 324 intergenic regions of the generic neural genes had tissue-specific ATAC-peaks in all 4 neural tissues, as shown in **Fig. 5c** (right). 172 intergenic regions of the generic neural genes had tissue-specific ATAC-peaks in the 3 neural tissues, as shown in **Fig. 5c** (left). Only 40 intergenic regions of the generic neural genes had tissue-specific ATAC-seq peaks in one neural tissue (Supplementary Methods).

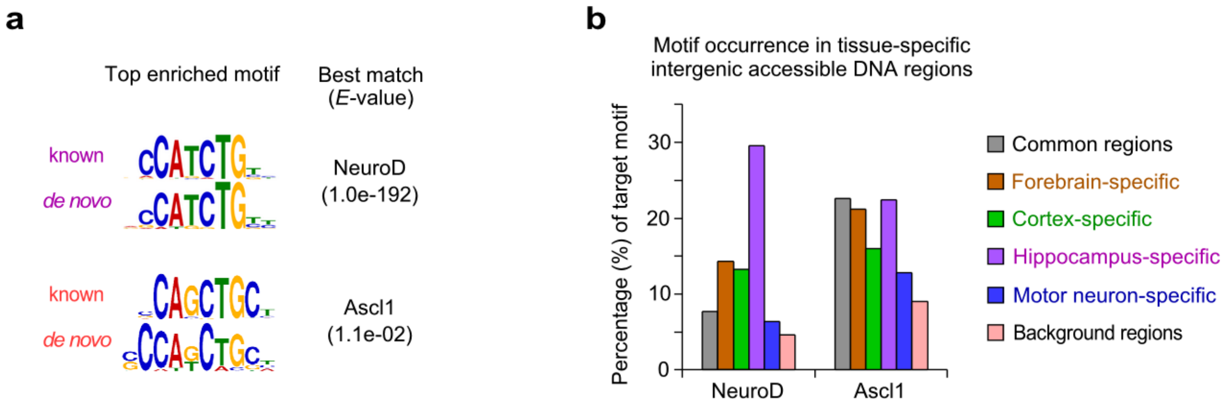

Supplementary Fig 7 The Ascl1 motif was found in the differentially accessible DNA sites in the multiple neural tissues.

a The *de novo* DNA motif search using MEME identified Ascl1 TF motif (bottom) in 4 neural tissues, shown in **Fig. 5**, then compared against a database of known motifs (Supplementary Methods). NeuroD TF motif (top) was identified from ATAC-seq peaks specific in the hippocampus (**Fig. 5b**). The *de novo* DNA motifs represent the most enriched motif within 50 bp from the midpoint of ATAC-seq peaks in each tissue.

b Percentage of the occurrence of the DNA motifs for the Ascl1 and NeuroD TFs within 100 bp from the midpoints of the tissue-specific intergenic ATAC-seq peaks, identified in **Fig. 5b** and background genomic regions (Supplementary Methods). The MEME motif format files used here were reported in Supplementary Data 11.

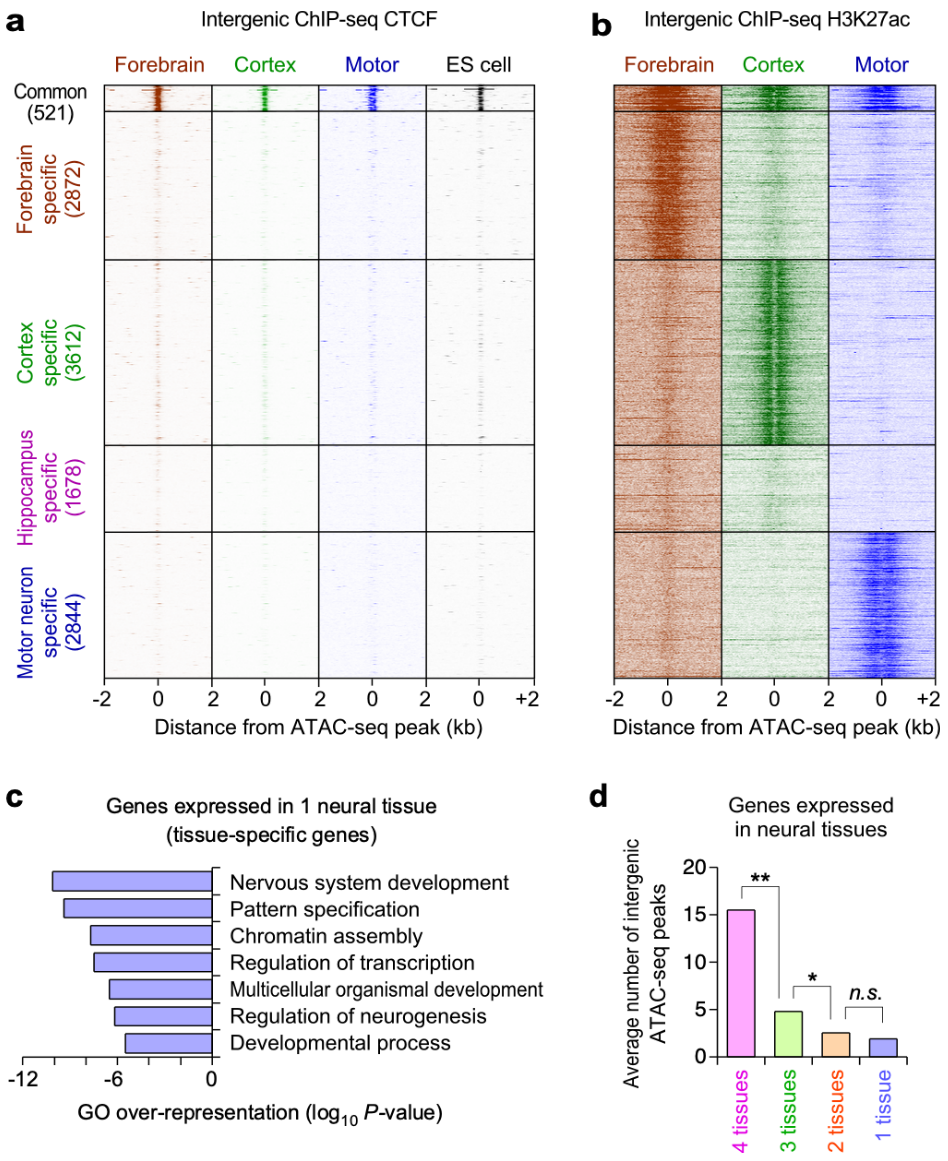

Supplementary Fig 8 The ATAC-seq peaks specific in each tissue were enriched with H3K27ac in a tissue-specific manner.

a, **b** ChIP-seq mapping of intergenic chromatin accessibility of 625 generic neural genes with >100 kb intergenic regions in 4 neural tissues, grouped as shown in **Fig. 5b**. 11,525 ATAC-peaks were sorted in the same order as shown in **Fig. 5b**. ChIP-seq CTCF signal was enriched in commonly accessible DNA sites (521 sites) in 4 neural tissues. Acetylation of histone H3K27 was enriched in tissue-specific ATAC-seq peaks in 3 neural tissues (the P0 forebrain, adult cortex, and embryonic motor neurons, Supplementary Data 12).

c Gene ontology analysis for the genes (1,330 genes) expressed in any 1 neural tissue out of 4 tissues (the P0 forebrain, adult cortex, adult hippocampus, or embryonic motor neurons), shown in **Fig. 5**. The most enriched seven non-redundant GO terms are shown. A one-sided Fisher's exact test was used to calculate *P*-values in gene set enrichment analysis. *P*-values were not adjusted for multiple comparisons. All GO target genes and background genes used for this GO analysis are listed in Supplementary Data 6.

d Average number of the ATAC-peaks in the intergenic regions of neural genes expressed in 1-4 neural tissues shown in **Fig. 5f** (4 tissues: 546 genes, 3 tissues: 666 genes, 2 tissues: 611 genes, 1 tissue: 1330 genes). The tissue-specific ATAC-seq peaks (11,004 peaks) that were identified in **Fig. 5b** were used. $^{**}P = 1.71 \times 10^{-22}$; $^{*}P = 3.51 \times 10^{-4}$; *n.s.*, non-significant $P = 0.17$; two-sided, two-sample *t*-test.

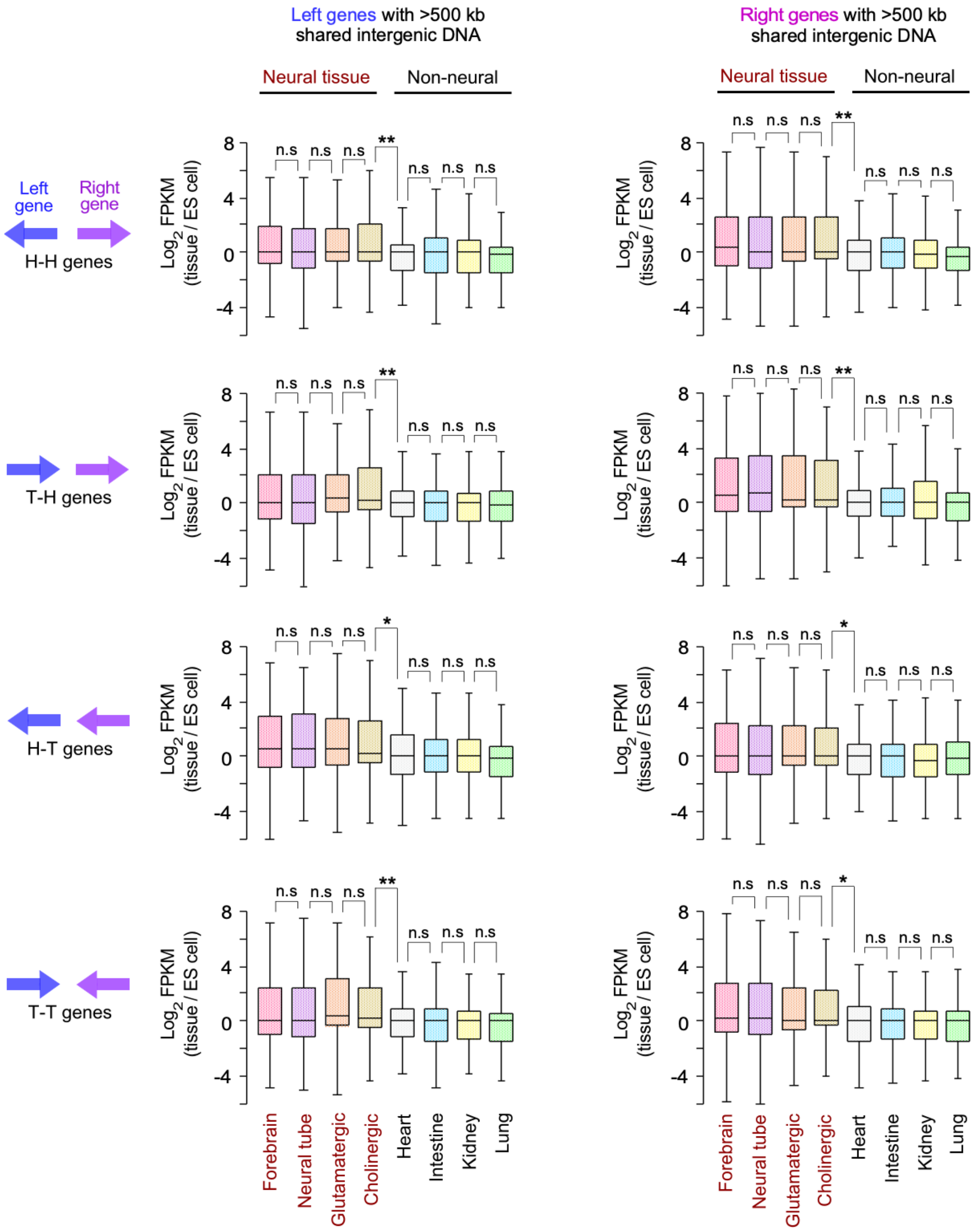

Supplementary Fig. 9 Extended intergenic DNA is associated with the expression of neighboring genes in neural tissues.

Box plots of gene induction levels of the left and right genes with >500 kb of shared intergenic DNA for each tissue in the 4 groups of the gene pairs, shown in **Fig. 6**. 4,196 H-H genes, 5,021 T-H genes, 5,031 H-T genes, and 3,792 T-T genes were used for the box plots. The box plots show the median (line in a box), first-to-third quartiles (boxes), and 1.5 × the interquartile range (whiskers). $^{**}P < 9.82 \times 10^{-4}$; $^{*}P < 1.65 \times 10^{-2}$; n.s., non-significant $P > 0.11$; two-sided, two-sample *t*-test (Supplementary Data 13).

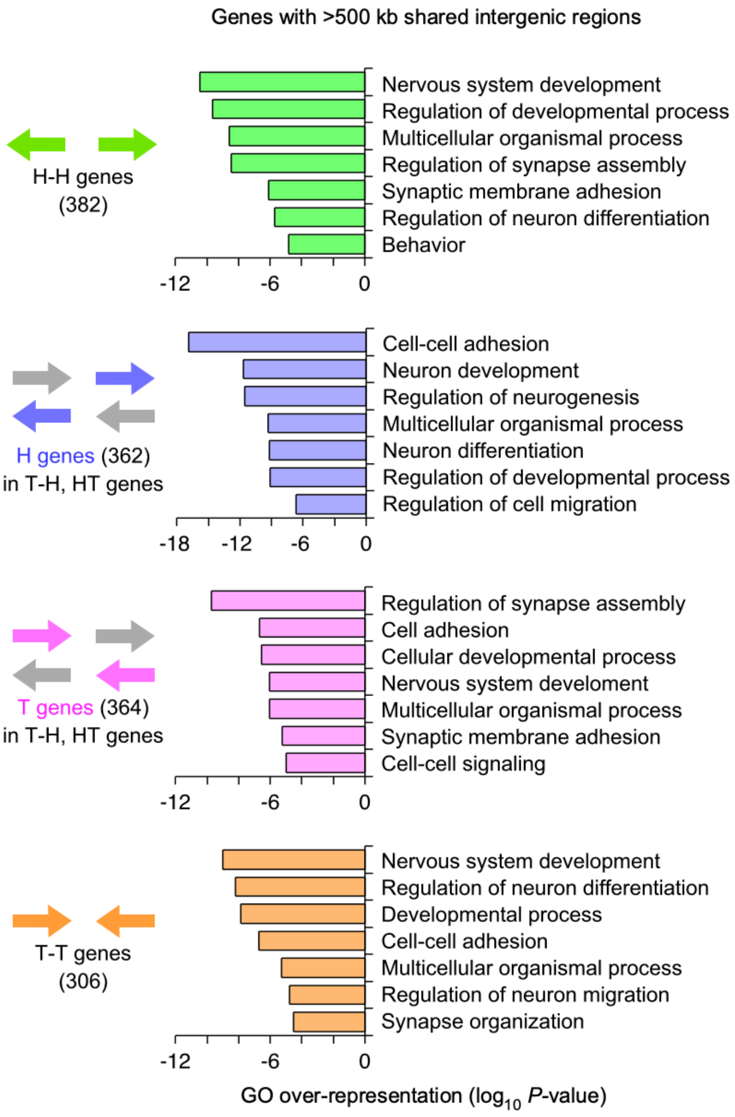

Supplementary Fig. 10 Neighboring genes with extended intergenic regions are enriched in neural gene annotations in all 4 groups of gene pairs.

Gene ontology analysis for the neighboring genes in the 4 groups of gene pairs shown in **Fig. 6**. The neighboring genes that share extended intergenic regions (>500 kb) were used in each group. The genes having the shared intergenic regions at the 5’ end of genes (H genes) and those having the shared intergenic regions at the 3’ end of genes (T genes) were illustrated as blue and pink arrows, respectively. The most enriched seven non-redundant GO terms are shown. A one-sided Fisher’s exact test was used to calculate *P*-values in gene set enrichment analysis. *P*-values were not adjusted for multiple comparisons. All GO target genes and background genes used for this GO analysis are listed in Supplementary Data 6.

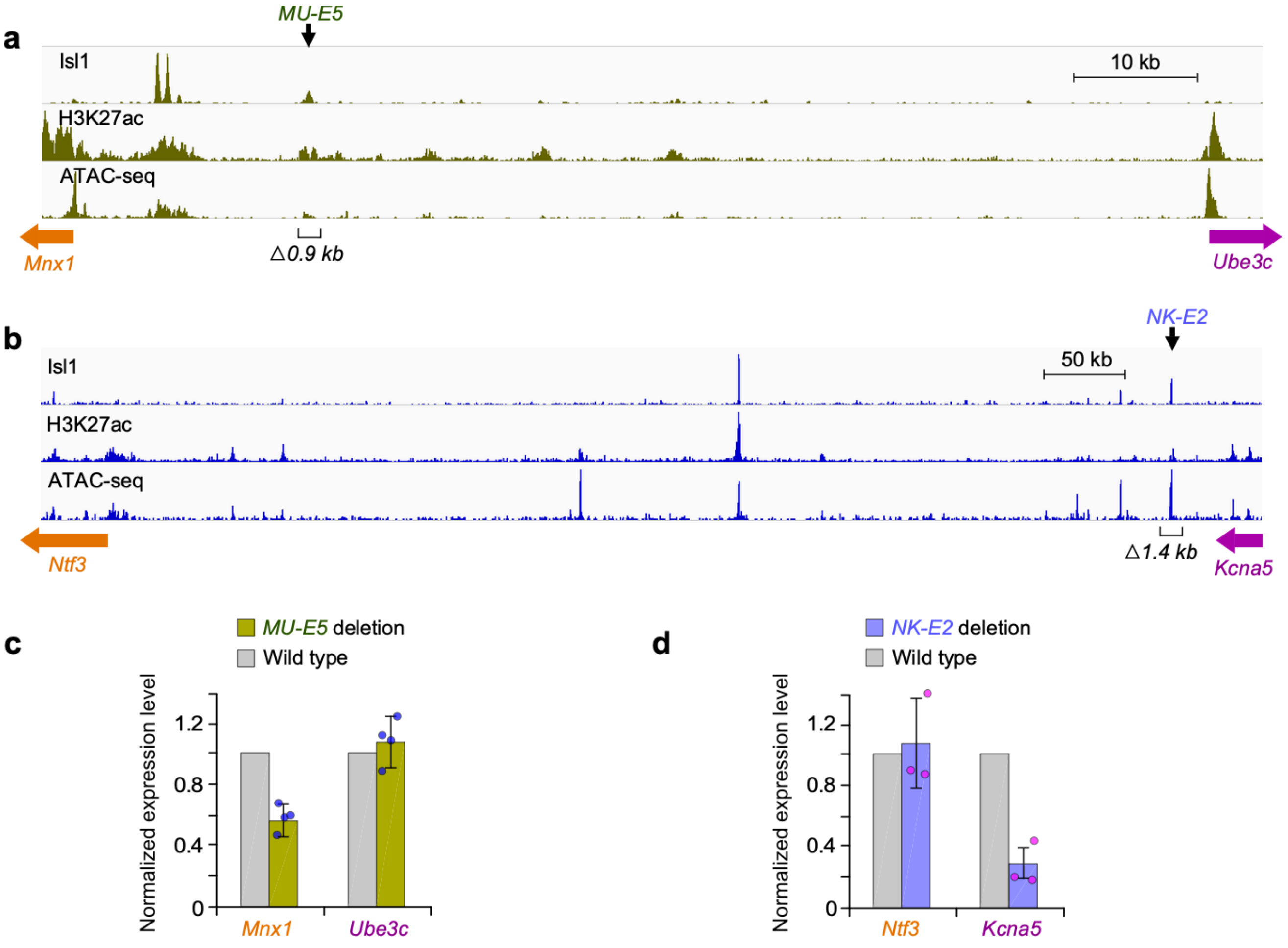

Supplementary Fig. 11 Intergenic enhancers contribute expression of neighboring genes in motor neurons.

a The location of Isl1-bound intergenic enhancers, detected by ChIP-seq Isl1, ChIP-seq H3K27ac, and ATAC-seq, located between *Mnx1* and *Ube3c* genes in motor neurons differentiated from mouse ES cells. 874 bp genomic DNA of motor neuron-specific enhancer (arrow, MU-E5) was deleted in an ES cell using CRISPR genome editing.

b Same as (**a**) except enhancers located in the intergenic region between *Ntf3* and *Kcna5* genes. 1,430 bp of the NK-E2 enhancer was deleted.

c Expression levels of neighboring genes, *Mnx1* and *Ube3c*, measured by quantitative RT-PCR in motor neurons containing the MU-E5 deletion, normalized to wild type expression levels of *Mnx1* and *Ube3c*. Error bars represent standard deviation (SD). Data are presented as mean values +/-SD. Dots represent the corresponding data points (Supplementary Data 14). 2 biologically independent differentiated cells were examined over 2 independent experiments ($n = 4$).

d Same as (**c**) except expression levels of *Ntf3* and *Kcna5* genes for NK-E2 enhancer deletion. 3 biologically independent differentiated cells were examined ($n = 3$).

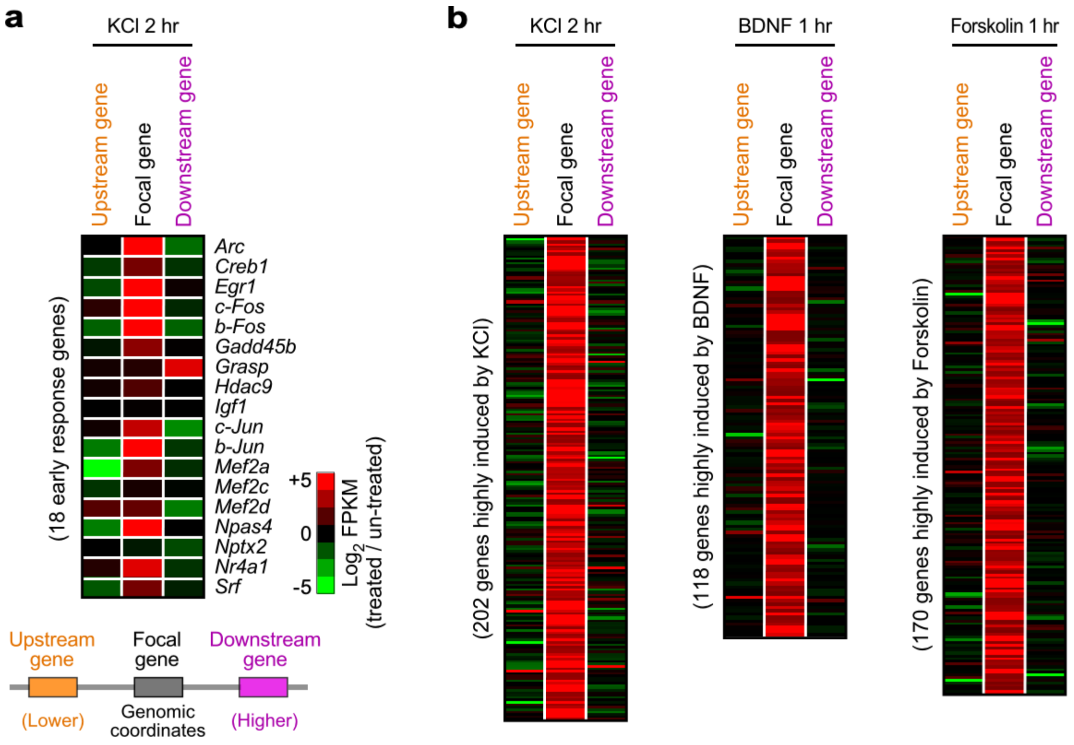

Supplementary Fig. 12 Most genes flanked by activity-dependent genes were not co-activated upon stimulation by KCl, BDNF, and forskolin.

a Heatmaps show the gene induction levels of the upstream, focal, and downstream genes, illustrated below the plot, after 2-hour KCl stimulation (log_2 fold-change in FPKM in treated / untreated neurons). Both treated and untreated neurons were incubated with tetrodotoxin (TTX) overnight to block sodium channels before KCL treatment. 18 early response genes, stimulated by KCl, are shown (Supplementary Data 16, 17).

b Same as (**a**) except showing genes highly induced by KCl, BDNF, and forskolin stimulation. 202 genes were highly induced by KCl stimulation (>2-fold compared to TTX treated neurons). Treated and untreated neurons were not incubated with TTX before stimulation by BDNF and forskolin for 1 hour. 118 genes were highly induced by BDNF stimulation (>1.5-fold compared to non-treated). 170 genes were highly induced by forskolin stimulation were shown (>1.5-fold compared to non-treated. Genes are sorted by a gene name (smallest to largest, Supplementary Data 16, 17).

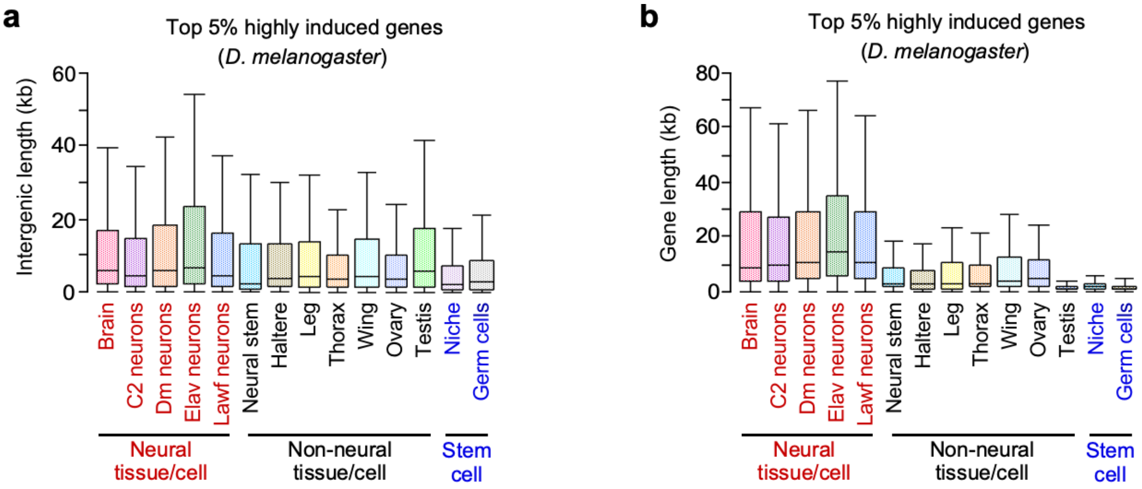

Supplementary Fig. 13 Highly induced genes in *Drosophila* neural tissues have significantly longer gene lengths than those in non-neural tissues.

a, b Box plots of intergenic DNA lengths (**a**) and gene lengths (**b**) of the top 5% of all the mRNA genes, highly induced in the fly tissues and cells shown in the shaded area in **Figs. 8c**, **e** (495 genes, Supplementary Data 3), compared to S2 cells. The box plots show the median (line in a box), first-to-third quartiles (boxes), and 1.5 × the interquartile range (whiskers).

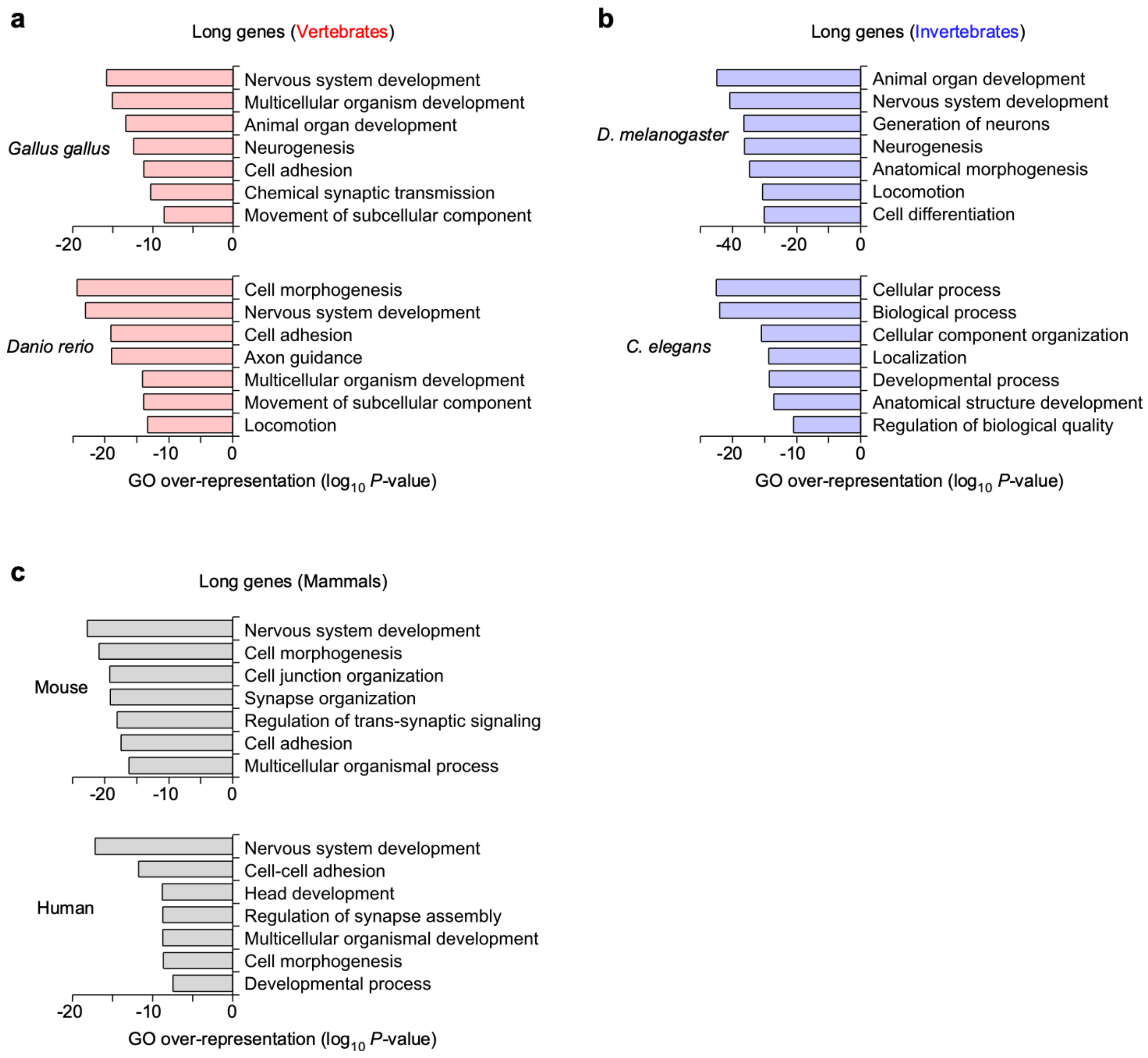

Supplementary Fig. 14 Long genes in vertebrates and *D. melanogaster* are enriched with neural gene annotations.

Gene ontology analysis for the top 5% of genes, having the longest gene lengths, of all the protein-coding genes in each multicellular organism. Invertebrates (**a**) include *D. melanogaster* and *C. elegans*. Vertebrates (**b**) include *Gallus gallus* and *Danio rerio*. Mammals (**c**) include *Mus musculus* and *Homo sapiens*. The most enriched seven non-redundant GO terms are shown (Supplementary Methods). A one-sided binomial test was used to calculate *P*-values in gene set enrichment analysis. *P*-values were not adjusted for multiple comparisons. All GO target genes and background genes used for this GO analysis are listed in Supplementary Data 6.

Supplementary Methods

Experimental Methods

Cell culture. The mouse embryonic stem (ES) cell line was derived from the inbred mouse strain 129/Ola ES-E14 male embryo. ES cells were cultured and differentiated into spinal motor neurons as described previously[1]. Briefly, ES cells plated on 0.1% gelatin-coated plate in EmbryoMax DMEM (Millipore, SLM220) supplemented with 10% ES cell-grade fetal bovine serum (Invitrogen, 12483020), L-glutamine (Gibco, 25030081), 0.1 mM beta-mercaptoethanol, and leukemia inhibitory factor (Millipore, ESG1107). After two days of the ES cell culture, we trypsinized the ES cells (Thermo, 25300054) and seeded them at 5 x 10^4 cells per mL in ADFNK medium (Advanced DMEM/F12:Neurobasal (1:1) Medium, 10% Knockout Serum Replacement, 2 mM L-glutamine, and 0.1 mM 2-mercaptoethanol) to initiate the formation of embryoid bodies (day 0). The ADFNK medium was exchanged on days 2 and 5 of differentiation. Patterning of embryoid bodies was induced by supplementing media on day 2 with 1 μM all-trans retinoic acid (Sigma, R2625) and 0.25 μM Smoothened agonist (SMO) of hedgehog signaling (Selleckchem, S7779). 5 μM DAPT (Selleckchem, S2215) was added to the ADFNK medium (10% knockout serum replacement, 2 mM L-glutamine, 0.1 mM β-mercaptoethanol in 1:1 ratio of Advanced DMEM/F12 (Gibco, 12634028) and Neurobasal medium (Gibco, 21103049)) on day 4 and day 5. Differentiated motor neurons were collected on day 6 and used for the downstream experiments such as RNA-seq and ChIP-seq as described below.

Generation of ESC lines containing enhancer deletion. The embryonic stem cell (ESC) line was derived from the inbred mouse strain 129/Ola ES-E14 male embryo. The RNA-guided Cas9 nuclease from the microbial Clustered Regularly Interspaced Short Palindromic Repeats (CRISPR/Cas9) system was used to delete enhancers in ESCs. The CRISPR/Cas9 genome editing was processed as described in Ran et al.[2] with some modifications. Briefly, guide RNAs (gRNAs) were designed with the available software at CRISPOR (http://crispor.tefor.net/crispor.py) and CHOPCHOP (https://chopchop.cbu.uib.no/). A pair of oligonucleotides encoding the 22-23 bp guide sequences containing 5'-NGG PAM (protospacer adjacent motif) sequence (underlined) to target the left and right sides of intended deletion of enhancers.

gRNA sequences to target the 1,719 bp region of the Ntf3-Kcna5-E1 enhancer (chromosome 6):

Forward 5' ATGCTCCTCTAAGACTTGGT<u>AGG</u> 3' (reverse/negative strand)

Reverse 5' GGGTGCCCACTACTTGTGCG<u>TGG</u> 3' (forward/positive strand)

gRNA sequences to target the 1,109 bp region of the Oprl1-Myt1-E1 enhancer (chromosome 2):

Forward 5' GCACAAAGCGTCAAACACCA<u>AGG</u> 3' (reverse/negative strand)

Reverse 5' GACTGGCTAGGTATCTTCAA<u>GGG</u> 3' (forward/positive strand)

gRNA sequences to target the 1,468 bp region of the Chrm2-Ptn-E1 enhancer (chromosome 6):

Forward 5' GCGTAGATGAATTCCTTGGG<u>AGG</u> 3' (forward/positive strand)

Reverse 5' TCTCCCACTAAGGAACACAG<u>AGG</u> 3' (reverse/negative strand)

gRNA sequences to target the 847 bp region of the Mnx1-Ube3c-E5 enhancer (chromosome 6):

Forward 5' TAGATGAGAGGTAGTAACCCAGG 3' (reverse/negative strand)

Reverse 5' GGACTTGGCATACAACCTAGTGG 3' (forward/positive strand)

gRNA sequences to target the 1,430 bp region of the Ntf3-Kcna5-E2 enhancer (chromosome 6):

Forward 5' TATGAGTGAGTAGCTATAGGTGG 3' (forward/positive strand)

Reverse 5' TTGGCAATGACTCGGGCATCAGG 3' (reverse/negative strand)

To synthesize gRNAs, we cloned 22-23 bp genomic sites of the form 5'-$N_{19\text{-}20}$NGG-3' (N=A, T, G, or C) near the intended target enhancer into U6 target gRNA expression vector (pgRNA vector) containing U6 promoter (Addgene, Ca # 41824) by following the available cloning protocol at https://www.addgene.org/static/cms/files/hCRISPR_gRNA_Synthesis.pdf. gRNA sequences were synthesized from IDT (Integrated DNA Technologies). To generate ESC lines containing enhancer deletion, ESCs were seeded and incubated for 48 hours on mouse embryonic fibroblast cells (EmbryoMax, PMEF-N) to maintain stem cell pluripotency. Cells were transiently transfected with a pair of pgRNA vectors and pCas9-GFP plasmid (Addgene, 44719) containing a CAGGS promoter with Cas9 DNA endonuclease and GFP reporter using Mouse P3 Primary Cell Nucleofector Kit (Lonza, VPG-1004) and Lonza Amaxa 4D-Nucleofector, according to the manufacturer's instructions. ESCs were dissociated 24-48 hours after transfection, GFP-expressing ESCs were selected by fluorescence-activated cell sorting (FACS). Approximately 2,000 sorted cells were plated on a dish with mouse feeder cells. ESCs formed clones in 4-6 days after FACS sorting and plating. A single colony was picked, followed by genotyping using PCR primers around genomic regions of intended enhancer deletion. The ESC clones containing enhancer deletion were sub-cultured to mouse feeder cells and then differentiated to postmitotic motor neurons as described previously[1].

Mouse embryos and cortical neuron stimulation.

Mouse studies were conducted strictly following all relevant ethical regulations in the animal use protocol (Protocol No. 20012209), which was approved by the Biological Sciences Local Animal Care Committee (LACC) at the University of Toronto, complying with guidelines established by the University of Toronto Animal Care Committee and the Canadian Council on Animal Care. All animal handling and experimental procedures were performed at the University of Toronto Mississauga Animal Facility. For mouse embryo studies, the C57BL/6 mouse strains (Mus musculus) were purchased from the Charles River Laboratories (Wilmington, MA, USA). Male C57BL/6 mice (aged 10 to 12 weeks) were mated with female mice (aged 8 to 10 weeks).

E16.5 embryonic mouse cortices were dissected from pregnant female mice, and then dissociated at 37°C for 20 min in papain dissociation solution containing HBSS (Gibco), 0.6 mg/mL papain, 0.5mM EDTA, 0.2 mg/mL L-cysteine (Sigma), 1.5 mM $CaCl_2$, 0.2 mg/mL DNase I (Sigma). Papain dissociation was stopped by adding 20% of horse serum (Gibco) and 80% of Neurobasal medium (Gibco) supplemented with B-27 (Gibco) and 0.5 mM glutamine (Gibco) for cortical neuron culture, as described in the previous studies[3,4] with some modifications. After gently dissociating the cortices by pipetting, tissues containing medium were filtered into a 50 mL tube with 40 µm cell strainer and then centrifuge at 80 x g for 5 minutes. The cells were resuspended with Neurobasal/B27 medium by gently pipetting. Dissociated neurons were plated on cell

culture dishes, pre-coated overnight with 20 μg/mL poly-d-lysine (Sigma) and 4 μg/mL laminin (Gibco). Before plating the cells, the dishes were washed twice with Neurobasal/B27 medium. Neurons were cultured in Neurobasal/B27 medium containing 0.5mM glutamine (Gibco) and penicillin/streptomycin (Gibco) at 37°C in a CO2 incubator (5%). 50% of the old medium was changed, and the cells were treated with 1 μM Ara-C (Sigma) on 3 days in vitro (DIV) and DIV5. For KCl stimulation, DIV5 cortical neurons were treated with 1 μM tetrodotoxin (TTX; Fisher) and 100 μM DL-AP5 (Fisher) overnight, and then changed into Neurobasal/B27 medium containing 31% of KCl depolarization buffer (170 mM KCl, 2 mM $CaCl_2$, 1 mM MgCl2, 10 mM HEPES) for 2 hr stimulation. For BDNF and forskolin stimulation, DIV6 neurons were treated with 10 ng/ml BDNF (R&D system) or 10 μM forskolin (Millipore) for 1 hr. For mouse breeding, C57BL/6 female and male mice were obtained from Charles River (stock #: 027C57BL/6). All animal experiments were approved by the University of Toronto Local Animal Care Committee (LACC).

RNA-seq. RNA-seq libraries were prepared as described previously with some modifications[1]. Briefly, total RNA for spinal motor neurons was extracted from day 6 of differentiated cells using TRIzol reagent (Thermo, 15596026). Total RNA for primary cortical neurons was extracted from 6 days in vitro culture using TRIzol reagent. NEBNext Poly(A) mRNA Magnetic Isolation Kit (NEB, E7490) was used to remove ribosomal RNA, followed by DNase treatment (Thermo, AM1906). RNA-seq libraries were prepared for sequencing using NEBNext RNA Library Prep Kit (NEB, E7765) and were sequenced by Illumina HiSeq 2500 for 50 bp single-end sequencing runs and Illumina NovaSeq 6000 System for 100 bp paired-end sequencing runs.

ChIP-seq. Approximately 25 million cells were crosslinked with 1% formaldehyde and then lysed. Chromatin pellets were isolated, solubilized, and then fragmented by sonication. Fragmented chromatin was then subjected to immunoprecipitation using magnetic beads coupled with Dynabeads Protein A (Invitrogen, 10001D, Lot# 00806319) and antibodies (~3 ug) against the protein of interest (H3K27ac; abcam, 4729, Lot# 3251519-2, CTCF; Millipore, 07-729, Lot# 3515588) in 1 mL of 0.5% bovine serum albumin in phosphate-buffered saline solution. After washing beads three times in ChIP wash buffer (500 mM sodium chloride, 50 mM HEPES, 1 mM EDTA, 1% Triton X-100, 0.1% sodium deoxycholate, 0.1% sodium dodecyl sulfate), ChIP-seq samples were eluted from the magnetic beads in ChIP elution buffer (1% sodium dodecyl sulfate, 10 mM EDTA), reverse crosslinked, and then purified. ChIP-seq libraries were prepared for sequencing using NEBNext DNA Library Prep Kit (NEB, E7645). Briefly, ChIP DNA was ligated with sequencing adapters, amplified, and gel purified. ChIP-seq libraries were sequenced by Illumina HiSeq 2500 for 50 bp single-end sequencing runs and Illumina Nova Seq 6000 for 100 bp paired-end sequencing runs.

Data Analyses

Datasets. Published mouse RNA-seq datasets were downloaded from the ENCODE project website (www.encodeproject.org), the Allen Brain Atlas data portal website (www.brain-map.org), and the previous studies[5-7]. Human RNA-seq datasets were obtained from the ENCODE project website and the previous

studies[8,9]. Relevant information about RNA-seq datasets including the Gene Expression Omnibus (GEO) accession numbers from the National Center for Biotechnology Information, used in each figure, are listed in Supplementary Data 1. Briefly, the spinal cord RNA-seq dataset was downloaded from the previous study[10]. Glutamatergic, GABAergic, and cholinergic neuron RNA-seq datasets were downloaded from the previous study[11]. Dorsal root ganglion and trigeminal neuron RNA-seq datasets were downloaded from the previous study[12]. Colonic sensory neuron RNA-seq dataset was downloaded from the previous study[13]. Single-cell (sc) RNA-seq data were treated as bulk RNA-seq data. In scRNA-seq data, the fragments per kilobase of transcript per million reads (FPKM) per gene were added for all the cells, then the sum of FPKM was divided by the number of cells.

Published ATAC-seq and ChIP-seq datasets were downloaded from the ENCODE project website and previous study[1]. Relevant information, including GSE accession numbers about these datasets, was listed in Supplementary Data 1. Briefly, ATAC-seq and H3K27ac ChIP-seq datasets in mouse tissues or cells (forebrain, neural tube, heart, intestine, kidney, lung, and ES cells) were downloaded from the ENCODE Project website[5]. ATAC-seq and H3K27ac ChIP-seq datasets in mouse motor neurons were downloaded from our previous study[1].

Processing of sequencing data. For next-generation sequencing data analysis, adapter sequences were removed using Trim Galore (version 0.4), downloaded from Babraham Bioinformatics Institute (http://www.bioinformatics.babraham.ac.uk/projects/trim_galore/). For RNA-seq analysis, the raw trimmed reads were aligned to the mouse reference genome using the STAR aligner (version 2.7), downloaded from GitHub (https://github.com/alexdobin/STAR)[14]. The gene annotation file (release M25, GRCm38.p6, comprehensive gene annotation file) was downloaded from GENCODE (www.gencodegenes.org). Then, fragments per kilobase of transcript per million reads (FPKM) were generated with the Cufflinks package (version 2.2.1), which was conducted using the Galaxy tools (https://usegalaxy.org/)[15]. For ATAC-seq or ChIP-seq analysis, the raw trimmed reads were aligned to the mouse genome with Bowtie2 aligner (version 2.4.2) using the Galaxy tools. Binding locations enriched by ChIP-seq H3K27ac were identified with the MACS2 peak caller (version 2.1.1) using Galaxy ChIP-seq tools. Published RNA-seq, ATAC-seq, ChIP-seq, and Hi-C datasets were downloaded from the ENCODE project website (www.encodeproject.org) and the previous studies, reported in Supplementary Data 1.

Defining genic and intergenic lengths. 19,144 of the annotated known mRNA genes (mouse mm9 NCBI Build 37 RefSeq Genes having complete coding start and end sequences) were used to define gene lengths and intergenic DNA lengths for most analyses except Hi-C analysis. Gene lengths were defined as the lengths between the transcription start site (TSS) and the transcription end site (TES) of all the annotated protein-coding genes. To define intergenic DNA lengths in the mouse Hi-C dataset, 21,269 of the annotated known mRNA genes were used (mouse mm10 NCBI RefSeq Genes having complete coding start and end sequences, Dec. 2011 assembly). To define intergenic DNA lengths in human datasets, 19,775 of the annotated protein-coding genes were used (hg19 Ensembl Genes, Feb. 2009 assembly). For *Drosophila*

datasets, 13,943 of the annotated known mRNA genes were used (*D. melanogaster* dm6 NCBI RefSeq Genes having complete coding start and end sequences, Aug. 2014 assembly).

The intergenic length of a gene was calculated as the sum of the upstream and downstream DNA distance from the end of a gene to the end of the nearest mRNA genes, as illustrated in **Fig. 3**. The ends of an mRNA gene were defined by the transcription start position (txStart) and the transcription end position (txEnd). The txStart of a gene was defined as the lower coordinate position among the TSS and the TES of a gene. The txEnd of a gene was defined as the upper coordinate position among the TSS and the TES of a gene. The upstream DNA distance was calculated by subtracting the txEnd of the upstream gene from the txStart of the downstream gene. The downstream DNA distance was calculated by subtracting the txEnd of the downstream gene from the txStart of the upstream gene. We could not calculate the intergenic lengths of genes located at the end of chromosomes, or both the txStart and txEnd of a gene reside within another gene. If one of the txStart or txEnd of a gene resides within another gene, we considered the intergenic length of the gene as the sum of the DNA distance from the gene to the end of the next non-overlapped mRNA gene. After filtering out these genes, we calculated and used the intergenic lengths for the following genes having the non-overlapped intergenic regions and the FPKM values: 18,383 mRNA genes (mm9), 19,857 mRNA genes (mm10) in mice, and 19,268 mRNA genes in humans (hg19).

Intergenic lengths of all the protein-coding genes in chickens, zebrafishes, insects, and nematodes were calculated as described above. Here we used 16,751 of all the annotated protein-coding genes in *Gallus gallus* (GRCg6a/galGal6 RefSeq genes, Mar. 2019 assembly), 25,289 of all the annotated protein-coding genes were used in *Danio rerio* (GRCz11/danRer11 RefSeq genes, May. 2017 assembly), 13,944 of all the annotated protein-coding genes in *Drosophila melanogaster* (BDGP Release 6/dm6, RefSeq genes, Aug. 2014 assembly), and 19,221 of all the annotated protein-coding genes in *Caenorhabditis elegans* (WBcel235/ce11, RefSeq genes, Feb. 2013 assembly). For **Fig. 8**, we used 9,897 mRNA genes, having the FPKM values in all 14 examined tissues and cells in *D. melanogaster*, for the intergenic length and gene length analyses. Intergenic lengths of all mRNA genes in mice, humans, and *D. melanogaster* are listed in Supplementary Data 2, 4, and 18, respectively. Gene lengths and intergenic lengths of all the protein-coding genes in *Gallus gallus*, *Danio rerio*, *D. melanogaster*, and *C. elegans* are listed in Supplementary Data 6.

Gene expression analysis. The FPKM values were used as the gene expression of an mRNA transcript, measured by RNA-seq. If multiple replicates of RNA-seq datasets are available, an average FPKM value of replicates was used per each mRNA gene. When comparing gene expression between multiple tissues, the FPKM values were median-normalized across all tissues using a median FPKM value of all mRNA genes per tissue as a normalizer. Some mRNA genes do not have the FPKM values in the processed RNA-seq datasets. Thus, the gene induction or expression data were filtered to include only the 18,383 mRNA genes that have the FPKM values and have non-overlapped intergenic regions. Prior to converting logarithmic scales, all the FPKM values were added by 0.1 to avoid producing no value in logarithmic scales with zero (log 0). Gene induction levels were calculated by the $\log_2$ fold-change in expression (FPKM) between the target gene (i.e., the gene in the tissue of interest) and the reference gene (i.e., the gene in the reference

tissue or cells). For example, gene induction levels (**Figs. 1, 2**) were calculated by $\log_2$ (tissue FPKM+0.1)/(ES cell FPKM+0.1). Absolute gene expression levels (**Fig. 3**) were calculated by $\log_{10}$ (FPKM+0.1) followed by median normalization. The heat map plots of mRNA expression levels were generated using Java Treeview 3.0 [16]. The FPKM values in heat map plots were median-normalized (median FPKM: 2) and $\log_2$ transformed.

To plot intergenic length against gene induction level (**Figs. 1, 2**), we use mean intergenic length followed by sorting genes from the highest to the lowest $\log_2$ fold-change in expression (bin size: 200 genes; 91 bins; 18,383 genes). Prior to sorting genes according to gene induction level, the order of all genes should be randomized using a "rand" function in Excel to avoid a spike around 0 value of $\log_2$ fold-change in expression. Otherwise, we observed a spike in mean gene induction plots around 0 value of $\log_2$ fold-change expression. We found that these spikes correspond to the olfactory or pheromone receptor genes (1,144 genes in the mouse genome). These genes have shorter intergenic lengths (median 39 kb), although they are neuronal genes. These genes are highly repressed (FPKM values: 0) in all other cell types and tissues except the olfactory or pheromone receptor neurons, as reported previously[17,18].

To plot gene induction or expression level against intergenic length (**Figs. 3, 6**), we sorted genes from the longest to the shortest intergenic length, then calculated the mean $\log_2$ fold-change in expression or the mean $\log_{10}$ expression within each bin (bin size: 200 genes, 91 bins). A moving average of 3 bins is used to smooth the irregularities of each bin. For these intergenic length plots, intergenic length (kb) on the x-axis was plotted as a logarithmic scale ($\log_{10}$ scale), then interpreted as a linear scale (non-log scale). For example, 1×10^3 kb of intergenic length on the x-axis was plotted as 3 ($\log_{10} 1 \times 10^3$), then described as 1×10^3 kb.

Box plot analysis. For the box plots of intergenic lengths of highly induced genes in each tissue and cell type (**Fig. 1**), data were filtered to include 18,383 mRNA genes that have the non-overlapped intergenic regions and the FPKM values. Thus, data exclude genes with negative intergenic lengths or genes with no FPKM values. Intergenic lengths (kb) of the top 5% of all mRNA genes, highly induced in each tissue or cell type (919 genes, Supplementary Data 2), were selected to generate the box plots (Supplementary Data 3). In the box and whisker plots, the ends of the box represent the lower (1st) and upper (3rd) quartiles, so the box spans the interquartile range. The median is marked by a line inside the box. The whiskers are the two lines outside the box that extend to 1.5x interquartile range as the highest and lowest observations. The box plot was generated using KaleidaGraph 4.5 (Synergy Software).

SNP analysis. To examine single nucleotide polymorphisms (SNPs) associated with Parkinson's disease (PD) genetic risk, we used the published SNP datasets for PD risk in humans (Pierce et al., 2018)[19]. This study reported a total of 23,918 SNPs associated with PD genetic risk (PD SNPs). 14,854 out of these 23,918 SNP nucleotides were located within 1 kb to each other. After excluding the SNPs within 1 kb, we identified 9,064 SNP locations (1 kb width). We further filtered out 4,604 SNPs that were located within genic regions of 19,268 mRNA genes, having non-overlapped intergenic regions in humans (hg19). After removing these genic SNPs, we identified 4,460 SNP locations to calculate their intergenic lengths and performed gene

ontology (GO) analysis of these SNP-associated genes. The GO analysis was performed as described in the below method section (GO analysis). The intergenic lengths containing these PD SNPs were determined as described above (Defining intergenic length).

Venn diagram analysis. In **Fig. 2**, we used all mRNA genes expressed (16,719 genes, FPKM > 0; Supplementary Data 2) in 3 CNS cell types (glutamatergic neurons, astrocytes, and oligodendrocytes), 3 non-neural tissues (the adult heart, adult intestine, and adult kidney), and ES cells in mice. First, we removed the housekeeping genes (755 genes). The housekeeping genes were defined as the genes highly induced in 4 neural tissues (the postnatal P0 forebrain, adult cortex, adult hippocampus, and embryonic motor neurons) and 4 non-neural tissues (the adult heart, adult intestine, adult kidney, and adult lung) in mice. Then, we select the top 10% of the most highly induced genes ($\log_2$ tissue/ES cell) out of all the expressed mRNA genes (1,596 out of 15,964 genes) in the 3 CNS cell types, followed by determination of the overlapping genes between the highly induced genes (1,596 genes) in each CNS tissue. The overlapped and un-overlapped genes shown in **Fig. 2** are listed in Supplementary Data 5.

Gene ontology (GO) analysis. GO term enrichment analyses in **Fig. 3**, Supplementary Figs. 3, 8, and 10 were performed using the Gene Ontology Enrichment Analysis and Visualization (GOrilla) system[20]. To remove redundant GO terms, we used the Reduce and Visualize Gene Ontology (REViGO) software with 0.7 of the SimRel similarity score based on Resnik's and Lin's similarity measures[21]. The top seven non-redundant GO terms were reported in the figures. In **Fig. 3**, all the annotated protein-coding genes except olfactory receptor genes were used as the background GO dataset (17,331 genes). Olfactory receptor genes were removed from the background GO datasets because several hundreds of highly paralogous olfactory receptor genes have very short gene lengths[18]. In Supplementary Figs. 3 and 8, all annotated genes expressed in at least 1 tissue or cell type, listed in each figure, were used as the background GO dataset. In Supplementary Fig. 10, all the mRNA genes defined with gene orientations were used as the background GO dataset.

For **Fig. 8** and Supplementary Fig 14, GO term enrichment analyses were performed using the PANTHER classification system v.14.0 (geneontology.org)[22] because the GOrilla does not support the GO analyses for some organisms examined in these figures, such as *Gallus gallus*. Default parameters were used for the GO overrepresentation test: GO terms that were enriched above the background with Binomial test. In **Fig. 8** and Supplementary Fig. 14, all the annotated unique genes in each organism were used as the background GO dataset. The GO terms from the GOrilla and PANTHER analyses were similar to each other. A full list of unfiltered GO terms (PANTHER) for **Figs. 3** and **8** were shown in Supplementary Data 7. All GO target genes and background genes for **Figs. 3, 8**, Supplementary Figs. 3, 8, 10, and 14 are listed in Supplementary Data 6.

Identifying ChIP-seq and ATAC-seq peaks. For the ChIP-seq and ATAC-seq datasets in motor neurons, peak calling was conducted as described previously[1]. Briefly, ChIP-seq peaks (binding events) enriched by

H3K27ac in motor neurons over background were identified using the strand separate peak calling algorithms using the GeneTrack software ("-s 50 -e 1000")[23,24]. The midpoint of the start and end position of the H3K27ac-enriched peaks was used as a peak coordinate. Genomic regions enriched by accessible DNA (ATAC-seq peaks) were identified using the MACS2 software[25]. For the published ChIP-seq and ATAC-seq datasets from the ENCODE datasets, we used the processed peak calls as H3K27ac-enriched or DNA-accessible regions. Active enhancers were defined as H3K27ac-enriched peaks whose genomic regions are located more than 3 kb from the nearest TSS of known mRNA genes (19,144 of the annotated RefSeq mRNA genes). The number of peak calls from ATAC-seq and ChIP-seq datasets was listed in Supplementary Data 8.

Distance of closest intergenic enhancers. To calculate the distance between the closest active enhancers per intergenic DNA in Supplementary Fig. 5, the top 5% of all mRNA genes, highly induced in each tissue and cell type (919 out of 18,383 genes), were used. Although we defined the intergenic length of a gene as the sum of the upstream and downstream DNA distance from the end of a gene to the nearest genes, here we considered intergenic DNA as each upstream and downstream DNA, separately, from the end of a gene to the nearest gene. Thus, we exclude extremely long distances between closest active enhancers due to a long genic DNA between two enhancers. Active enhancers were defined as H3K27ac peaks determined by ChIP-seq. The distance between enhancers was considered when two or more enhancers exist in an intergenic DNA. Less than 1 kb of the distances between enhancers were filtered out because two enhancers within 1 kb were considered as a single enhancer.

Motif enrichment analysis. We used the Multiple Em for Motif Elicitation (MEME, version 5.4.1) algorithm for the *de novo* motif discovery[26]. We searched the enriched motifs occurring proximal to accessible DNA regions within 50 bp from the midpoints of the identified ATAC-seq peaks in the mouse P0 forebrain, adult cortex, adult hippocampus, and embryonic stem cell-derived motor neuron (Supplementary Data 1). The top enriched the *de novo* DNA motifs with the most significant *E*-values, relative to the genomic background in each tissue-specific accessible DNA region, were used in **Fig. 5**. *E*-values in MEME show the significance of the motif based on the Markov background model. For MEME, 6–12 bp width of motifs were searched. The motif sites were expected to be distributed zero or one occurrence per sequence with the 0-order background sequence model. The known DNA motifs matched with the *de novo* DNA motifs were searched against a transcription factor binding DNA motif database (JASPAR CORE 2022, non-redundant vertebrate motifs *in vivo* and *in silico*)[27] using the motif comparison tool Tomtom (version 5.4.1)[28], which ranks the DNA motifs in the database and generates an alignment for each significant matched DNA motif in the database. For the Tomtom analysis, the motifs were compared using the Pearson correlation coefficient with *E*-value < 1.0e-10. The top-matched motifs with the most significant *P*-value were reported.

For the target motif occurrence analysis, we used the Find Individual Motif Occurrences (FIMO, version 5.4.1)[29]. For the FIMO analysis, we used Minimal MEME Motif Format, which is a plain format containing the DNA motif letters, background letter frequencies, and letter-probability matrix. 100 bp from the midpoints of

the identified ATAC-seq peaks in **Fig. 5** were searched for the occurrence of the target motifs on both DNA strands with the matched *P*-value < 1.0e-05. To get background genomic regions, we randomly selected 1,436 genomic coordinates throughout the mouse genome (mm10), then extracted genomic DNA sequences within 100 bp from the random genomic coordinates. All Minimal MEME Motif Formats (zip file) for the target DNA motifs identified in **Fig. 5** and Supplementary Fig. 7 are available in Supplementary Data 11.

Hi-C analysis. To examine long-range chromatin interactions between intergenic enhancers and TSS of the gene in neural cells and tissues, we used the published Hi-C dataset for mouse cortical neurons (Bonev et al., 2017)[30]. We used mm10 (GRCm38) mouse assembly and gene annotation for the Hi-C analysis. This study (Bonev et al., 2017) reported a total of 18,094 interactions between enhancers and TSSs in cortical neurons. Among them, we selected 16,429 interactions involved with protein-coding genes, then identified 8,238 interactions that are associated with intergenic enhancers. 2,214 out of 8,238 interactions were involved with genes with long intergenic DNA regions (>500 kb, LID genes). We then examined interactions between intergenic enhancers and LID genes in cortical neurons. Most enhancers interact with multiple TSSs, and we found that 1,254 enhancers were involved in 2,214 interactions with TSSs of 818 genes. Neighboring genes of these 818 genes were identified as described above (Neighboring gene analysis). All Hi-C interactions, enhancers, and genes involved in this Hi-C analysis were reported in Supplementary Data 15.

Neighboring gene analysis. To calculate the shared intergenic DNA length of neighboring genes, we considered gene orientations and transcription directions of two neighboring genes. We classified all the mouse mRNA genes (mm10; 18,040 genes) into 4 groups based on the orientation of neighboring gene pairs: head-to-head (H-H) genes where the two genes were located on the opposite DNA strands and transcribed divergently; tail-to-head (T-H) genes where the two genes were located on the upper DNA strand and transcribed in the same direction; head-to-tail (H-T) genes where the two genes were located on the lower DNA strand and transcribed in the same direction; and tail-to-tail (T-T) genes where the two genes are located on the opposite DNA strands and transcribed convergently (**Fig. 6**). Then, we define a left gene and a right gene, which are located at lower and higher genomic coordinates of the shared intergenic DNA region, respectively, in each group of gene pairs. A left gene shares an intergenic region with a right gene on the right side of the left gene. All left and right neighboring genes and their shared intergenic lengths are listed in Supplementary Data 13. The heatmap plots for induction levels of neighboring genes ($\log_2$ FPKM expression in tissue/ES cells) were generated using Java TreeView[16].

Significance test. The comparison of intergenic DNA lengths or the number of peaks of highly induced genes between tissues or cell types is reported as *P*-value heatmap plots. The *P*-values for the difference in intergenic DNA length or the number of peaks between tissues were calculated based on a Student's *t*-distribution under the null hypothesis. We used the null hypothesis because we expect no significant difference between two populations or between certain characteristics of a population. We used a two-sample *t*-test with the Excel function "*t*-test" with tails=2 (two-tailed distribution) and type=2 (homoscedastic equal

variance). Intergenic lengths (kb) of the top 5% of all mRNA genes, highly induced in each tissue (919 genes) and the *P*-values for their differences (**Figs. 1, 8**) are listed in Supplementary Data 3. The *P*-values for the difference in the number of ATAC-seq peaks and ChIP-seq peaks (**Fig. 4**) between pairwise combinations of tissues or cell types used in the heatmap plots are listed in Supplementary Data 3.

Supplementary References

1 Rhee, H. S. *et al.* Expression of Terminal Effector Genes in Mammalian Neurons Is Maintained by a Dynamic Relay of Transient Enhancers. *Neuron* **92**, 1252-1265, doi:10.1016/j.neuron.2016.11.037 (2016).

2 Ran, F. A. *et al.* Genome engineering using the CRISPR-Cas9 system. *Nat Protoc* **8**, 2281-2308, doi:10.1038/nprot.2013.143 (2013).

3 Malik, A. N. *et al.* Genome-wide identification and characterization of functional neuronal activity-dependent enhancers. *Nat Neurosci* **17**, 1330-1339, doi:10.1038/nn.3808 (2014).

4 Joo, J. Y., Schaukowitch, K., Farbiak, L., Kilaru, G. & Kim, T. K. Stimulus-specific combinatorial functionality of neuronal c-fos enhancers. *Nat Neurosci* **19**, 75-83, doi:10.1038/nn.4170 (2016).

5 Shen, Y. *et al.* A map of the cis-regulatory sequences in the mouse genome. *Nature* **488**, 116-120, doi:10.1038/nature11243 (2012).

6 Yue, F. *et al.* A comparative encyclopedia of DNA elements in the mouse genome. *Nature* **515**, 355-364, doi:10.1038/nature13992 (2014).

7 Djebali, S. *et al.* Landscape of transcription in human cells. *Nature* **489**, 101-108, doi:10.1038/nature11233 (2012).

8 Gray, J. M. *et al.* SnapShot-Seq: a method for extracting genome-wide, in vivo mRNA dynamics from a single total RNA sample. *PLoS One* **9**, e89673, doi:10.1371/journal.pone.0089673 (2014).

9 Consortium, E. P. An integrated encyclopedia of DNA elements in the human genome. *Nature* **489**, 57-74, doi:10.1038/nature11247 (2012).

10 Chen, K. *et al.* RNA-seq characterization of spinal cord injury transcriptome in acute/subacute phases: a resource for understanding the pathology at the systems level. *PLoS One* **8**, e72567, doi:10.1371/journal.pone.0072567 (2013).

11 Tasic, B. *et al.* Adult mouse cortical cell taxonomy revealed by single cell transcriptomics. *Nat Neurosci* **19**, 335-346, doi:10.1038/nn.4216 (2016).

12 Lopes, D. M., Denk, F. & McMahon, S. B. The Molecular Fingerprint of Dorsal Root and Trigeminal Ganglion Neurons. *Front Mol Neurosci* **10**, 304, doi:10.3389/fnmol.2017.00304 (2017).

13 Hockley, J. R. F. *et al.* Single-cell RNAseq reveals seven classes of colonic sensory neuron. *Gut* **68**, 633-644, doi:10.1136/gutjnl-2017-315631 (2019).

14 Dobin, A. *et al.* STAR: ultrafast universal RNA-seq aligner. *Bioinformatics* **29**, 15-21, doi:10.1093/bioinformatics/bts635 (2013).

15 Afgan, E. *et al.* The Galaxy platform for accessible, reproducible and collaborative biomedical analyses: 2018 update. *Nucleic Acids Res* **46**, W537-W544, doi:10.1093/nar/gky379 (2018).

16 Saldanha, A. J. Java Treeview--extensible visualization of microarray data. *Bioinformatics* **20**, 3246-3248, doi:10.1093/bioinformatics/bth349 (2004).

17 Gabel, H. W. *et al.* Disruption of DNA-methylation-dependent long gene repression in Rett syndrome. *Nature* **522**, 89-93, doi:10.1038/nature14319 (2015).

18 Magklara, A. & Lomvardas, S. Stochastic gene expression in mammals: lessons from olfaction. *Trends Cell Biol* **23**, 449-456, doi:10.1016/j.tcb.2013.04.005 (2013).

19 Pierce, S. E., Tyson, T., Booms, A., Prahl, J. & Coetzee, G. A. Parkinson's disease genetic risk in a midbrain neuronal cell line. *Neurobiol Dis* **114**, 53-64, doi:10.1016/j.nbd.2018.02.007 (2018).

20 Eden, E., Navon, R., Steinfeld, I., Lipson, D. & Yakhini, Z. GOrilla: a tool for discovery and visualization of enriched GO terms in ranked gene lists. *BMC Bioinformatics* **10**, 48, doi:10.1186/1471-2105-10-48 (2009).

21 Supek, F., Bosnjak, M., Skunca, N. & Smuc, T. REVIGO summarizes and visualizes long lists of gene ontology terms. *PLoS One* **6**, e21800, doi:10.1371/journal.pone.0021800 (2011).

22 Mi, H., Muruganujan, A., Ebert, D., Huang, X. & Thomas, P. D. PANTHER version 14: more genomes, a new PANTHER GO-slim and improvements in enrichment analysis tools. *Nucleic Acids Res* **47**, D419-D426, doi:10.1093/nar/gky1038 (2019).

23 Albert, I., Wachi, S., Jiang, C. & Pugh, B. F. GeneTrack--a genomic data processing and visualization framework. *Bioinformatics* **24**, 1305-1306, doi:10.1093/bioinformatics/btn119 (2008).

24 Rhee, H. S. & Pugh, B. F. Comprehensive genome-wide protein-DNA interactions detected at single-nucleotide resolution. *Cell* **147**, 1408-1419, doi:10.1016/j.cell.2011.11.013 (2011).

25 Feng, J., Liu, T., Qin, B., Zhang, Y. & Liu, X. S. Identifying ChIP-seq enrichment using MACS. *Nat Protoc* **7**, 1728-1740, doi:10.1038/nprot.2012.101 (2012).

26 Bailey, T. L., Johnson, J., Grant, C. E. & Noble, W. S. The MEME Suite. *Nucleic Acids Res* **43**, W39-49, doi:10.1093/nar/gkv416 (2015).

27 Stormo, G. D. Modeling the specificity of protein-DNA interactions. *Quant Biol* **1**, 115-130, doi:10.1007/s40484-013-0012-4 (2013).

28 Gupta, S., Stamatoyannopoulos, J. A., Bailey, T. L. & Noble, W. S. Quantifying similarity between motifs. *Genome Biol* **8**, R24, doi:10.1186/gb-2007-8-2-r24 (2007).

29 Grant, C. E., Bailey, T. L. & Noble, W. S. FIMO: scanning for occurrences of a given motif. *Bioinformatics* **27**, 1017-1018, doi:10.1093/bioinformatics/btr064 (2011).

30 Bonev, B. *et al.* Multiscale 3D Genome Rewiring during Mouse Neural Development. *Cell* **171**, 557-572 e524, doi:10.1016/j.cell.2017.09.043 (2017).
