# Supplementary material for: Extended intergenic DNA contributes to neuron-specific expression of neighboring genes in the mammalian nervous system
Source: Nat Commun. 2022 May 18;13:2733. doi: 10.1038/s41467-022-30192-z (PMC9117226; doi:10.1038/s41467-022-30192-z)
Supplement: Supplementary file 21 — Reporting Summary [file 41467_2022_30192_MOESM21_ESM.pdf]

nature portfolio

Corresponding author(s): Ho Sung Rhee

Last updated by author(s): Mar 20, 2022

Reporting Summary

Nature Portfolio wishes to improve the reproducibility of the work that we publish. This form provides structure for consistency and transparency in reporting. For further information on Nature Portfolio policies, see our Editorial Policies and the Editorial Policy Checklist.

Statistics

For all statistical analyses, confirm that the following items are present in the figure legend, table legend, main text, or Methods section.

n/a	Confirmed	
☐	☒	The exact sample size (*n*) for each experimental group/condition, given as a discrete number and unit of measurement
☐	☒	A statement on whether measurements were taken from distinct samples or whether the same sample was measured repeatedly
☐	☒	The statistical test(s) used AND whether they are one- or two-sided<br>*Only common tests should be described solely by name; describe more complex techniques in the Methods section.*
☒	☐	A description of all covariates tested
☐	☒	A description of any assumptions or corrections, such as tests of normality and adjustment for multiple comparisons
☐	☒	A full description of the statistical parameters including central tendency (e.g. means) or other basic estimates (e.g. regression coefficient) AND variation (e.g. standard deviation) or associated estimates of uncertainty (e.g. confidence intervals)
☐	☒	For null hypothesis testing, the test statistic (e.g. *F*, *t*, *r*) with confidence intervals, effect sizes, degrees of freedom and *P* value noted<br>*Give P values as exact values whenever suitable.*
☒	☐	For Bayesian analysis, information on the choice of priors and Markov chain Monte Carlo settings
☒	☐	For hierarchical and complex designs, identification of the appropriate level for tests and full reporting of outcomes
☐	☒	Estimates of effect sizes (e.g. Cohen's *d*, Pearson's *r*), indicating how they were calculated

Our web collection on statistics for biologists contains articles on many of the points above.

Software and code

Policy information about availability of computer code

Data collection

No software was used to collect the data in this study.

Data analysis

The following open-source software packages were used for data analysis. For the next-generation sequencing data analysis, FASTQC (version 0.11.9, https://www.bioinformatics.babraham.ac.uk/projects/fastqc/) was run to check the sequencing quality. Adapter sequences were removed using Trim Galore (version 0.4), downloaded from Babraham Bioinformatics Institute (http://www.bioinformatics.babraham.ac.uk/projects/trim_galore/). For RNA-seq analysis, the raw trimmed reads were aligned to the mouse reference genome using the STAR aligner (version 2.7), downloaded from GitHub (https://github.com/alexdobin/STAR). The gene annotation file (release M25, GRCm38.p6, comprehensive gene annotation file) was downloaded from GENCODE (www.gencodegenes.org). Then, fragments per kilobase of transcript per million reads (FPKM) were generated with the Cufflinks package (version 2.2.1), which was conducted using the Galaxy tools (https://usegalaxy.org/). For ATAC-seq or ChIP-seq analysis, the raw trimmed reads were aligned to the mouse genome with Bowtie2 aligner (version 2.4.2) using the Galaxy tools. Binding locations enriched by ChIP-seq H3K27ac were identified with the MACS2 peak caller (version 2.1.1) using Galaxy tools.

For gene ontology (GO) analysis, GO term enrichment analyses were performed using the Gene Ontology Enrichment Analysis and Visualization system (version 2013Mar, http://cbl-gorilla.cs.technion.ac.il/) and the PANTHER classification system (version 14.0, http://geneontology.org/). To remove redundant GO terms, we used the Reduce and Visualize Gene Ontology software (version 2021Nov, http://revigo.irb.hr/). The box plot was generated using KaleidaGraph (version 4.5, Synergy Software, Reading, PA, USA).

For motif enrichment analysis, we used the Multiple Em for Motif Elicitation (MEME, version 5.4.1) algorithm for the de novo motif discovery (https://meme-suite.org/meme/tools/meme). We searched the 6–12 bp width of enriched motifs occurring proximal to accessible DNA regions within 50 bp from the midpoints of the identified ATAC-seq peaks. The known DNA motifs matched with the de novo DNA motifs were searched against a transcription factor binding DNA motif database (JASPAR CORE, version 2022, https://jaspar.genereg.net/) using the motif

comparison tool Tomtom (version 5.4.1, https://meme-suite.org/meme/tools/tomtom). For the target motif occurrence analysis, we used the Find Individual Motif Occurrences (FIMO, version 5.4.1, https://meme-suite.org/meme/tools/fimo).

For manuscripts utilizing custom algorithms or software that are central to the research but not yet described in published literature, software must be made available to editors and reviewers. We strongly encourage code deposition in a community repository (e.g. GitHub). See the Nature Portfolio guidelines for submitting code & software for further information.

Data

Policy information about availability of data

All manuscripts must include a data availability statement. This statement should provide the following information, where applicable:

- Accession codes, unique identifiers, or web links for publicly available datasets
- A description of any restrictions on data availability
- For clinical datasets or third party data, please ensure that the statement adheres to our policy

The RNA-seq datasets for cortical neuron experiments have been deposited in the National Center for Biotechnology Information (NCBI) Sequence Read Archive (SRA) under the study number SRP272183. The RNA-seq and ChIP-seq datasets for motor neuron experiments have been deposited in the NCBI Gene Expression Omnibus (GEO) under the accession number GSE154532 and GSE196170.

Field-specific reporting

Please select the one below that is the best fit for your research. If you are not sure, read the appropriate sections before making your selection.

☒ Life sciences ☐ Behavioural & social sciences ☐ Ecological, evolutionary & environmental sciences

For a reference copy of the document with all sections, see nature.com/documents/nr-reporting-summary-flat.pdf

Life sciences study design

All studies must disclose on these points even when the disclosure is negative.

Sample size	Sample sizes for RNA-seq, ATAC-seq, and ChIP-seq have been determined empirically to provide statistically sufficient datasets for analysis in this study. Statistical methods were not used to predetermine sample size. For RNA-seq, ChIP-seq, and ATAC-seq experiments, we used two independent biological replicates except Fig. 4a, Supplementary Figs. 8a, and 12. For enhancer deletion experiments in Fig. 7 and Supplementary Fig. 11, at least two independent biological replicates per genotype were used.
Data exclusions	No datasets were excluded for the analysis.
Replication	All data presented in this study are derived from at least two independent biological replicates except Fig. 4a which as from one replicate of ATAC-seq or DNase-seq, Supplementary Fig. 8 which was from one ChIP-seq replicate, and Supplementary Fig. 12 which was from one replicate of the mouse cortical neuron stimulation experiment. To verify the reproducibility of motor neuron differentiation experiments for RNA-seq, ChIP-seq, and enhancer deletion experiments, we stained differentiated cells with motor neuron-specific antibody Hb9 and performed quantitative RT-PCR. All experiments with replicates were repeated independently and reproduced successfully.
Randomization	Randomization was used for the experiments involving mouse embryos. Randomization was not applied to in vitro cell line experiments, because the covariates in motor neuron differentiation are not relevant due to the same genetic background of our in vitro cell lines and known genotypes under the same cell culture condition. Thus, randomization was not feasible given motor neuron differentiation derived from mouse embryonic stems and genomic data production here.
Blinding	Blinding was not feasible given mouse embryonic stem cell-derived differentiation and genomic data production here, because all experiments are controlled by genotypes and processed simultaneously. Thus, blinding was not applied to the experiments in this study, based on the objective nature of the measurements.

Reporting for specific materials, systems and methods

We require information from authors about some types of materials, experimental systems and methods used in many studies. Here, indicate whether each material, system or method listed is relevant to your study. If you are not sure if a list item applies to your research, read the appropriate section before selecting a response.

Materials & experimental systems

n/a	Involved in the study	
☒	☐	Antibodies
☐	☒	Eukaryotic cell lines
☒	☐	Palaeontology and archaeology
☐	☒	Animals and other organisms
☒	☐	Human research participants
☒	☐	Clinical data
☒	☐	Dual use research of concern

Methods

n/a	Involved in the study	
☐	☒	ChIP-seq
☒	☐	Flow cytometry
☒	☐	MRI-based neuroimaging

Eukaryotic cell lines

Policy information about cell lines

Cell line source(s)	The mouse embryonic stem (ES) cell line was derived from the inbred mouse strain 129/Ola ES-E14 male embryo. To maintain ES cell pluripotency, mouse embryonic fibroblast (MEF feeder) cells were purchased from MilliporeSigma (Burlington, MA, USA; EmbryoMax, Cat No. PMEF-N).
Authentication	During cell culture, parental lines were authenticated based on the monitoring of phenotypic features (ES cell morphology, embryoid body formation, medium color, etc.). Characteristics of the ES cell line and differentiated motor neurons were previously reported by previous studies. Immunostaining experiments were performed to confirm the expression of molecular markers during motor neuron differentiation.
Mycoplasma contamination	All cell lines were tested negative for mycoplasma contamination.
Commonly misidentified lines (See ICLAC register)	No commonly misidentified cell lines were used in this study.

Animals and other organisms

Policy information about studies involving animals; ARRIVE guidelines recommended for reporting animal research

Laboratory animals	To collect E16.5 mouse embryos, the C57BL/6 mouse strains (Mus musculus) were purchased from the Charles River Laboratories (Wilmington, MA, USA). Male C57BL/6 mice (aged 10 to 12 weeks) were mated with female mice (aged 8 to 10 weeks). Mice were bred and maintained at 19-23 °C with 40-60% humidity on a fixed 12-hour light and 12-hour dark cycle in which lights on at 7 am and lights off at 7 pm.
Wild animals	No wild animals used in this study.
Field-collected samples	No field-collected samples were used in this study.
Ethics oversight	Mouse studies were conducted strictly following all relevant ethical regulations in the animal use protocol (Protocol No. 20012209), which was approved by the Biological Sciences Local Animal Care Committee (LACC) at the University of Toronto, complying with guidelines established by the University of Toronto Animal Care Committee and the Canadian Council on Animal Care.

Note that full information on the approval of the study protocol must also be provided in the manuscript.

ChIP-seq

Data deposition

☒ Confirm that both raw and final processed data have been deposited in a public database such as GEO.

☒ Confirm that you have deposited or provided access to graph files (e.g. BED files) for the called peaks.

Data access links *May remain private before publication.*	The raw RNA-seq datasets for cortical neuron experiments have been deposited in the National Center for Biotechnology Information (NCBI) Sequence Read Archive (SRA) under the study number SRP272183. The processed RNA-seq datasets for cortical neuron experiments are included in Supplementary Data 16. The RNA-seq and ChIP-seq datasets (raw and processed) for motor neuron experiments have been deposited in the NCBI Gene Expression Omnibus (GEO) under the accession number GSE154532 and GSE196170.
Files in database submission	7 RNA-seq database files and 4 ChIP-seq data files were generated from this study were submitted to NCBI GEO and SRA. A list of the database files used in this study were reported by previous studies.<br>RNA-seq spinal motor neurons - WT rep1 (SRX8742596)<br>RNA-seq spinal motor neurons - WT rep2 (SRX8742597)<br>RNA-seq primary cortical neurons, DIV6, TTX treated (SRX11668067)<br>RNA-seq primary cortical neurons, DIV6, TTX, 2 hr KCl treated (SRX11668068)<br>RNA-seq primary cortical neurons, DIV6, non-treated (SRX11668080)<br>RNA-seq primary cortical neurons, DIV6, 1 hr BDNF treated (SRX11668277)

RNA-seq primary cortical neurons, DIV6, 1 hr forskolin treated (SRX11668365)
ChIP-seq spinal motor neurons - WT H3K27ac rep1 (SRX14046356)
ChIP-seq spinal motor neurons - WT H3K27ac rep2 (SRX14046357)
ChIP-seq spinal motor neurons - WT CTCF rep1 (SRX14046358)
ChIP-seq spinal motor neurons - WT no antibody rep1 (SRX14046355)

Genome browser session (e.g. UCSC)

No genome browser sessions were generated for this study.

Methodology

Replicates

For RNA-seq datasets, two independent biological replicates were used for spinal motor neurons, and one replicate was used for primary cortical neurons.

Sequencing depth

Length of reads, 50 bp, single-end reads for RNA-seq motor neuron and ChIP-seq H3K27ac datasets
Length of reads, 150 bp, paired-end reads for RNA-seq cortical neuron and ChIP-seq CTCF, no antiboy datasets
RNA-seq motor neuron rep1 total reads: 20,215,358
RNA-seq motor neuron rep1 uniquely mapped: 16,839,680
RNA-seq motor neuron rep2 total reads: 80,040,643
RNA-seq motor neuron rep2 uniquely mapped: 66,394,448
RNA-seq primary cortical neurons, DIV6, TTX total reads: 29,877,969
RNA-seq primary cortical neurons, DIV6, TTX uniquely mapped: 17,249,975
RNA-seq primary cortical neurons, DIV6, TTX, 2 hr KCl total reads: 42,960,473
RNA-seq primary cortical neurons, DIV6, TTX, 2 hr KCl uniquely mapped: 25,901,630
RNA-seq primary cortical neurons, DIV6, non-treated total reads: 45,080,161
RNA-seq primary cortical neurons, DIV6, non-treated uniquely mapped: 26,827,652
RNA-seq primary cortical neurons, DIV6, 1 hr BDNF total reads: 42,755,161
RNA-seq primary cortical neurons, DIV6, 1 hr BDNF uniquely mapped: 28,083,695
RNA-seq primary cortical neurons, DIV6, 1 hr forskolin total reads: 34,107,013
RNA-seq primary cortical neurons, DIV6, 1 hr forskolin uniquely mapped: 21,991,375
ChIP-seq spinal motor neurons - WT H3K27ac rep1 total reads: 14,283,342
ChIP-seq spinal motor neurons - WT H3K27ac rep1 uniquely mapped: 12,266,631
ChIP-seq spinal motor neurons - WT H3K27ac rep2 total reads: 36,237,534
ChIP-seq spinal motor neurons - WT H3K27ac rep2 uniquely mapped: 33,770,719
ChIP-seq spinal motor neurons - WT CTCF rep1 total reads: 20,180,276
ChIP-seq spinal motor neurons - WT CTCF rep1 uniquely mapped: 6,630,967
ChIP-seq spinal motor neurons - WT no antibody rep1 total reads: 19,983,585
ChIP-seq spinal motor neurons - WT no antibody rep1 uniquely mapped: 9,864,617

Antibodies

H3K27ac (abcam, 4729, Lot# 3251519-2) and CTCF (Millipore, 07-729, Lot# 3515588)

Peak calling parameters

Binding locations enriched by ChIP-seq H3K27ac were identified with the MACS2 peak caller (version 2.1.1) using Galaxy tools. The following parameter setting was used for peak calling: MFOLD range: 5-50, band width: 300, peaks based on: q-value, minimum FDR: 0.05.

Data quality

FASTQC (version 0.11.9, https://www.bioinformatics.babraham.ac.uk/projects/fastqc/) is run to check the sequencing quality.

Software

FASTQC (version 0.11.9); Trim Galore (version 0.4); STAR aligner (version 2.7); Cufflinks (version 2.2.1); Bowtie2 aligner (version 2.4.2); MACS2 peak caller (version 2.1.1)
